# Childhood maltreatment and clinical response to mood stabilizers in patients with bipolar disorder

**DOI:** 10.1192/j.eurpsy.2023.1204

**Published:** 2023-07-19

**Authors:** N. Attianese, S. Donato, M. Battipaglia, R. Ceres, G. D’Agostino, G. Cascino

**Affiliations:** 1 University “L.Vanvitelli”, Napoli; 2 University of Salerno, Salerno, Italy

## Abstract

**Introduction:**

Childhood maltreatment (CM) is recognized to be a non-specific risk factor for the development of psychiatric disorders in adulthood. It has been consistently demonstrated that exposure to CM increases the risk of developing bipolar disorder (BD). In addition, CM has been associated with worse clinical presentation and course of BD. CM has been also linked to poorer responses to psychotropic drug treatments in different psychiatric disorders.

**Objectives:**

The aim of the current study was to explore retrospectively the impact of CM on the response to prophylactic treatment with lithium or anticonvulsants in a cohort of adult BD patients. Based on the reported literature, we hypothesized that BD patients with a history of CM would present a poorer response to both lithium and anticonvulsant treatments.

**Methods:**

Participants were recruited from patients consecutively attending the outpatient facilities of the Psychiatric Unit of the University of Salerno. The following inclusion criteria were adopted: (1) diagnosis of BD type 1 or type 2 according to DSM‐5 criteria; (2) age ≥ 18 years; (3) willingness to participate in the study, expressed by written informed consent; (4) stable adequate treatments with mood stabilizers (at least 1‐year duration and, in the case of lithium, at therapeutic blood levels); (5) being clinically euthymic at the time of inclusion. Retrospective treatment response was evaluated by using the Alda scale. CM history was assessed by means of the short form of the Childhood Trauma Questionnaire (CTQ).

**Results:**

Thirty‐seven patients (24 with a history of CM and 13 without CM) were on stable lithium treatment while sixty (35 with a history of CM and 25 without CM) were on stable anticonvulsant treatment. Clinical response to drug treatment did not differ between BD with CM and those without CM in the whole sample as well as in the anticonvulsant‐treated subgroup. In the lithium‐treated subgroup, a significant negative correlation emerged between physical abuse and treatment response (ρ = −0.38; p = 0.03) and patients with CM showed a significantly reduced Alda score (p = 0.04).

**Conclusions:**

In patients with BD, CM did not influence the clinical response of anticonvulsants, whereas it was associated with a poorer response to lithium with childhood physical abuse playing a major role in this effect.

**Disclosure of Interest:**

None Declared

